# Spirometry reference values in the Brazilian population

**DOI:** 10.1590/1414-431X20175700

**Published:** 2017-03-02

**Authors:** R. Rufino, C.H. Costa, A.J. Lopes, A.I. Maiworm, K. Maynard, L.M.R.A. Silva, R.M. Dias

**Affiliations:** 1 Departamento de Doenças do Tórax, Faculdade de Ciências Médicas, Universidade do Estado do Rio de Janeiro, Rio de Janeiro, RJ, Brasil; 2 Faculdade de Economia, Universidade Federal do Rio de Janeiro, Rio de Janeiro, RJ, Brasil; 3 Departamento Cardiopulmonar, Escola de Medicina e Cirurgia, Universidade Federal do Estado do Rio de Janeiro, Rio de Janeiro, RJ, Brasil

**Keywords:** Spirometry, Lung function test, Reference values, FVC, FEV_1_

## Abstract

The aim of the present study was to provide new spirometry reference equations in a sample of the Brazilian population for the following parameters: forced vital capacity (FVC), forced expiratory volume in 1 second (FEV_1_), FEV_1_/FVC ratio, peak of expiratory flow (PEF), forced expiratory flow at 50% (FEF_50%_)_,_ 75% average vital capacity (FEF_25-75%_), and average forced expiratory flow time (FEFT). This was a prospective study using results from chest radiographs, electrocardiograms, and questionnaires to investigate the participants' respiratory symptoms, sedentarism, and comorbidities (Charlson comorbidity index). From December 2010 to July 2014, individuals were randomly selected from various locations in the state of Rio de Janeiro. All individuals were examined by a single technician in the morning at the laboratory, and performed the spirometry with the same spirometer. Spirometry values were tabulated for the creation of three equation models: linear regression, logarithmic regression, and logarithms through a method that incorporates the lambda, median, and coefficient of variation (LMS method). Initially, 7003 individuals from both genders were contacted, and 454 were recruited. The data from the new equations were compared with one Brazilian and eight international equations, resulting in a high correlation (r>0.9). The values derived from the LMS method and linear regression were very similar (P>0.5), and both could be used to acquire the reference values for Brazilian spirometry. Data derived from the equations of this study were different from the current Brazilian equation, which could be justified by the different method used.

## Introduction

Spirometric measurements can be interpreted by comparing numbers derived from a certain population. Spirometry reference values (or standard numbers) are derived from healthy individuals (1). The reference population should represent the general population (2,3).

According to the Brazilian Institute of Geography and Statistics (IBGE - Instituto Brasileiro de Geografia e Estatística) (4), Brazil has had many spontaneous migratory flows since 1884, which continue into the present day. Germans, Spaniards, Italians, Japanese, Portuguese, Syrians, Turks, Africans, along with the native indigenous population constitute the Brazilian gene pool (4). Thus, Brazilian spirometric reference values could and should present characteristics similar to other populations around the world.

The aim of this study is to present an equation for the Brazilian population using an alternate method in order to validate the present reference values for spirometry (5), yield new numbers, or support data from international literature.

## Material and Methods

We conducted a cross-sectional study and evaluated healthy individuals (20 to 80 years old) with sedentary lifestyles from December 2010 to July 2014. The study was approved by the Research Ethics Committee of the Universidade do Estado do Rio de Janeiro (UERJ, 2782/2010-CAAE 0226.0.228.00-10). Volunteers were randomly selected from various regions of the state of Rio de Janeiro, in Southeastern Brazil. The locations chosen for pre-selection included the Sports Authority of Rio de Janeiro, Senior Citizens University, Herbert Viana Hemotherapy Unit, Greater Rio Samba Schools (Salgueiro, Vila Isabel, and Mangueira). Volunteers were undergraduate and graduate students from UERJ and Veiga de Almeida University, visitors of patients from Hospital Universitário Pedro Ernesto, and citizens from various neighborhoods in Rio de Janeiro. Ethnicity was self-defined.

Considering a Brazilian population of 190,000,000 at the time this research started in December of 2010 (IBGE) (4), the sample size was calculated to yield a 95% confidence level with a 5% margin of error. Despite the population growth to 204,761,379 (IBGE 2015) from 2011 to 2015, the calculated number of individuals remained the same. Therefore, in order to obtain a representative sample of the Brazilian population, 377 individuals were necessary. The percentage of individuals for each age group was calculated based on IBGE (4) data.

The inclusion criteria were sedentary individuals between 20 and 80 years of age, and absence of a smoking history, and pulmonary, cardiac, and neurological diseases. A sedentary lifestyle was defined as no or irregular physical activity less than 150 min a week according to the World Health Organization (6).

The exclusion criteria were smokers and ex-smokers, illegal drug users, and individuals with any of the following: active lifestyles; spirometry results not meeting the criteria for acceptability and reproducibility (2); radiographic evidence of pleuropulmonary lesions [including pulmonary masses, hyperinflation pattern (flattened hemidiaphragm) or interstitial disease]; specific abnormal findings on electrocardiograms such as ischemic region, myocardial infarction, tachyarrhythmia, or complete ventricular blockages; respiratory infections of the upper and/or lower airways in the 6 weeks before the spirometry; a Charlson index above 1 (7); cognitive deficit preventing comprehension of the questionnaire and implementation of the pulmonary function test (8); debilitating and chronic diseases, such as cardiopathies, pneumopathies, and neuropathies; unstable angina or arterial hypertension (systolic arterial pressure >200 mmHg or diastolic arterial pressure >110 mmHg) (2); a recent history of cardiac arrhythmia or myocardial infarction (2,3); medication use for treating cardiopathies (beta-blockers) (9); respiratory atopies (2,3,10); and hemoptysis.

All chest radiographs were analyzed by a pulmonologist holding a specialist title from the Brazilian Society of Pneumology and Tisiology (BSPT). The electrocardiogram was performed by the Cardiology Service. The physician responsible for the report was a cardiologist from the Brazilian Society of Cardiology (BSC). Physical therapists received prior training for the administration of the pre-selection questionnaire (2), the Charlson index (7), and the International Physical Activity Questionnaire (11).

### Spirometry

Spirometry exams were conducted from 8:00 am to 12:00 noon, at the Laboratory of Pulmonary Function of the UERJ, a referral center with 25 years of experience accredited by the BSPT for training professionals in pulmonary function in the state of Rio de Janeiro.

The exams followed the American Thoracic Society (ATS) 1987 protocol (12), which was adapted for the Brazilian Guidelines for Pulmonary Function Tests (2002) (2).

The device used was the Vitatrace VT 130 SL (Codax Ltda., Brazil) breath spirometer, which was integrated to the Spiromatic 2.0 program (Engelógica, Rio de Janeiro, Brazil). A maximum of 8 breaths (forced spirometry manipulations) and a minimum of 3 were taken in order to meet the acceptability and reproducibility criteria.

The acceptability criteria of the curves adhered to the following criteria proposed by ATS (12) and BSPT (2): a retroextrapolation volume <5% of the forced vital capacity (FVC) or 150 mL; forced expiration duration of at least 6 s; occurrence of a plateau in the volume-time curve for at least 1 s, after a minimum expiratory time of 6 s, or a volume in the last second lower than 25 mL, and a number of 3 to 8 breaths, with at least 3 acceptable and 2 reproducible curves.

The criteria for curve reproducibility followed those proposed by ATS/ERS (European Respiratory society) (3) and BSPT (2): difference in FVC in the best two curves <150 mL, forced expiratory volume in 1 s (FEV_1_) in the best two curves <150 mL, and difference in the peak of expiratory flow (PEF) in the best curves <10%.

The outcome variables were FVC, FEV_1_, FEV_1_/FVC, PEF, 25 to 75% forced expiratory flow from the curve (FEF_25-75%_), FEF_50%_, FEF_75%_, and average forced expiratory flow time (FEFT).

### Statistical analysis and derivation of equations

Kolmogorov-Smirnov tests were performed in order to determine whether the study population was homogeneous. Subsequently, parametric tests (Student's *t*-test and the Pearson correlation equation) were used to analyze values with normal distribution, taking into account the average and standard deviation. Anthropometric and spirometry data are reported as medians and percentages, using number and point graphs. All analyses were carried out in Stata 14 (StataCorp LP, USA). Estimated coefficients with P<0.05 were considered to be significant.

In the univariate regression analysis, the dependent variables were the spirometric indices. Correlation coefficient tests of the functional parameters with anthropometric variables and their transformations were conducted. Variables that had a P<0.10 were selected for inclusion in the multivariate analysis.

After determining the multiple regression equations, residuals were identified and their adherence to the normal curve was graphically confirmed. In addition, the asymmetry of the equation was analyzed by 4 tests: Mardia's asymmetric, Mardia's kurtosis, Henze-Zirkler, and Doornik-Hansen. The one with the greatest value was noted.

The residual method was used to establish the threshold of the reference value, once the regression equation was calculated. The calculated value was subtracted from the average residual value of the equation and corrected by the standard deviation, in order to achieve 95% confidence level (multiplied by the constant 1.645).

The following three equations were calculated for FVC and FEV_1_: multivariate linear regression, logarithmic regression, and logarithmic regression with the spline variable. This variable is being used for equations of the Global Lung Function Initiative (GLI) (13) and recent Japanese equations (14).

Logarithmic spline equations were calculated with the lambda-median-coefficient of variation (LMS) method. The purpose of the spline is to turn the dependent variable into a non-linear variable (from parametric to non-parametric), allowing it to be an exploratory parameter that can vary slightly (non-linearly). The spline variable was defined after finding the mean, deviation, and asymmetry of the equation, considering the standard normal distribution:



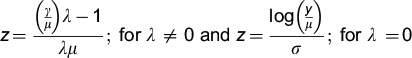
(1)where LMS (λ, μ, σ) = λ (asymmetry), μ (mean), σ (deviation).

After the transformation and assuming that the variable (y) features a smooth curve, the Box-Cox method was applied, with age interpolation via the Box-Cox Cole and Green methods.

Regression equations were calculated according to Pereira et al. (5), Knudson et al. (10), Crapo et al. (15), Hankinson et al. (16), Pérez et al. (17), Falaschetti et al. (18), and Brändli et al. (19) and compared with the equations of the present study (referred to as Rufino et al.). The values from Kubota et al. (14) and GLI (13) were compared using the spline method (or LMS). It was also used the Bland-Altman method with GraphPad Prism 6:04 (USA) for agreement analysis.

## Results

**Figure 1:**
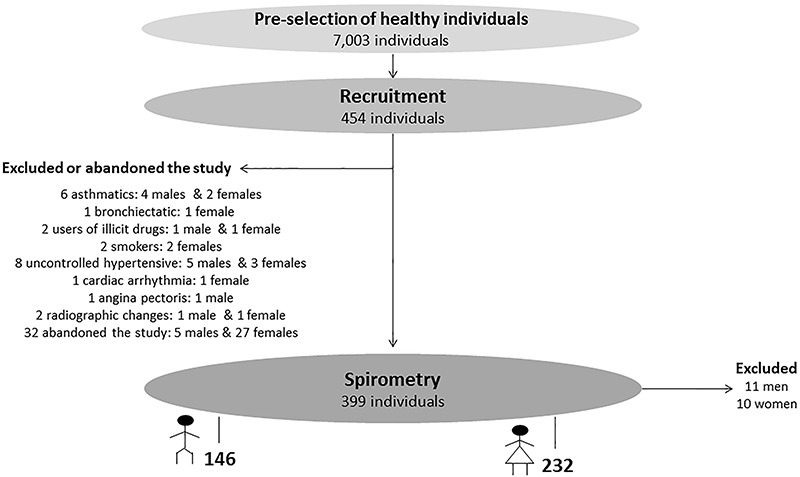
Patient recruitment flowchart. A total of 16.73% excluded or abandoned the study (12.11% during the test stage, 4.62% during spirometry); a total of 83.27% completed all phases.

**Figure 2:**
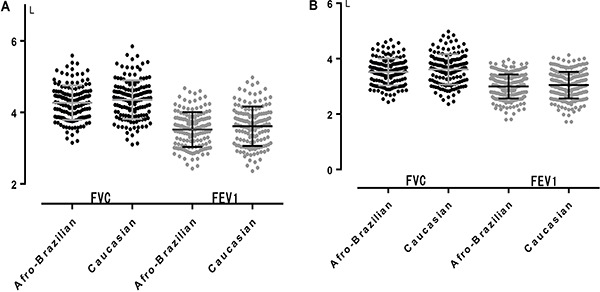
Forced vital capacity (FVC) and forced expiratory volume in 1 second (FEV1) in two Brazilian ethnicities. Horizontal lines indicate mean±SD.

From December of 2010 to July of 2014, a total of 454 individuals were recruited, with 55 being excluded (Figure 1). The sample showed homogeneity when grouped according to age and according to gender (Table 1). More women than men were recruited; this is due to the higher frequency of diseases among men, as well as a higher level of physical activity compared to women. The weight (in kg) was higher in men than in women. However, when corrected by height squared to calculate BMI, differences in weight were non-significant. The lack of significant differences between BMI and age demonstrated that male and female groups were homogeneous (Table 1). The age varied between 20 and 74 in males, and between 20 and 80 in females. There were no differences among age groups selected in 20-year intervals, and between African and non-African descent, based on self-defined ethnicity (Figure 2).

**Table:**
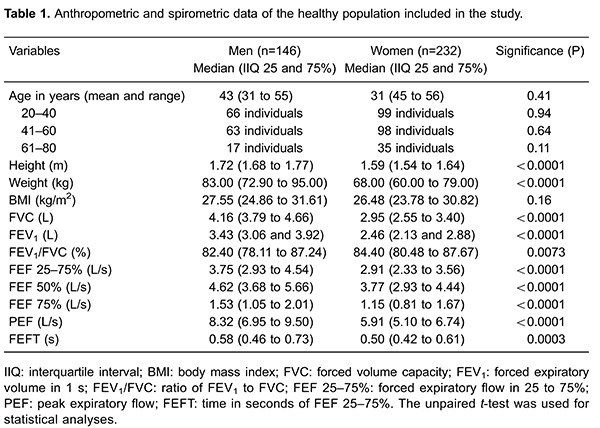

**Figure 3:**
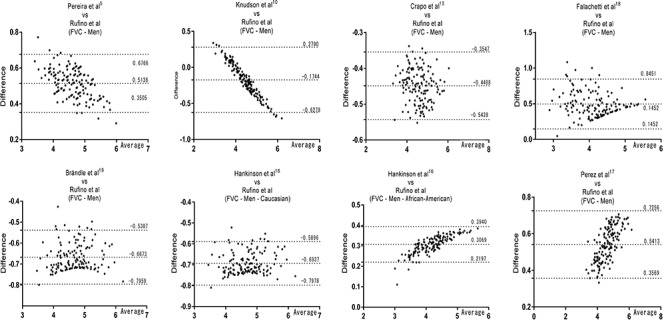
Results of the Bland-Altman test in the forced vital capacity (FVC) in men. Rufino et al. is the present study.

Except for FEV_1_/FVC ratio, spirometric indices were all higher in males than in females. There was a decrease of approximately 20 mL per year of age in FVC and FEV_1_ for both genders. The correlations of anthropometric variables were compared with spirometric indices and the r^2^ value was identified (Tables 2 and 3; Figure 3).

**Table:**
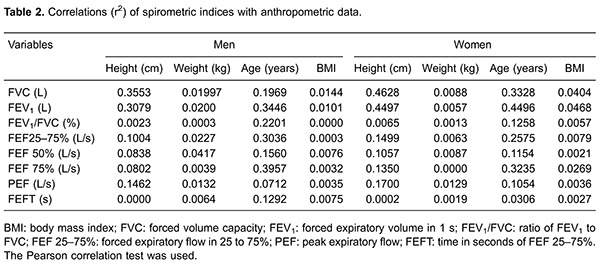

Equation coefficients for men and women are reported in Table 3. The logarithmic spline data calculated through the LMS method are reported in Table 4. Table 5 shows the correlations between the proposed equations and the other eight equations currently used worldwide. The non-significant differences between the findings obtained through the linear equation and the LMS (spline) equation are reported in Table 6.

**Table:**
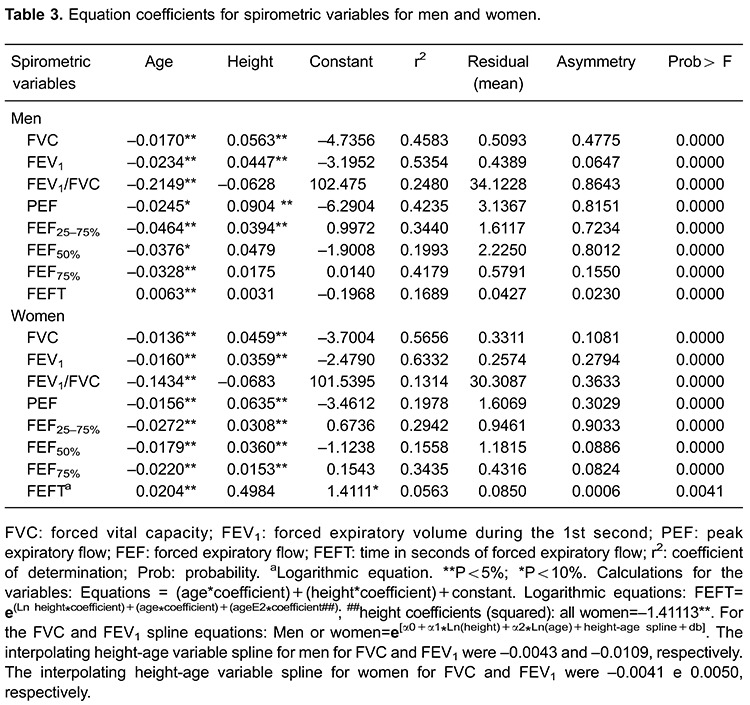

**Table:**
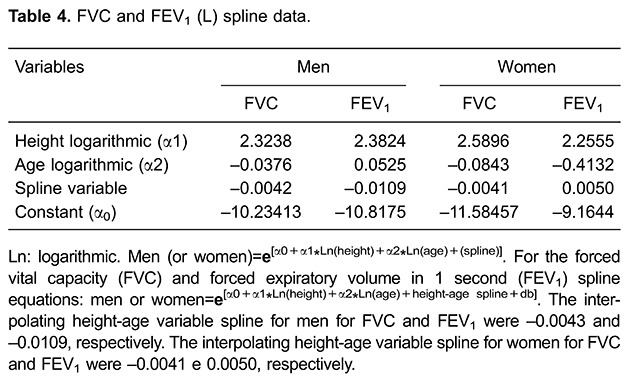

**Table:**
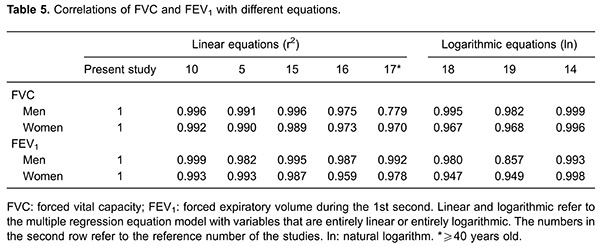

**Table:**
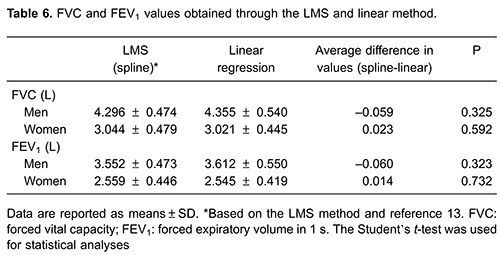

## Discussion

One of the most important tools in the sustainability of equations for reference values in a healthy population is the proper definition of health itself (1,20,21). Being "healthy" means not having a disease or having an effectively controlled disease. However, the drugs used for controlling diseases often change pulmonary function (e.g., beta-blockers) (9), which in turn decreases the pool of healthy people in lung analysis, as the frequency of diseases increases with age (22,23). In a Scottish study (23) involving 1,751,841 patients, a large proportion of individuals >60 had eight disorders. At the age of 85, all had at least one disorder and almost 10% had eight disorders, including systemic arterial hypertension, diabetes mellitus, osteoporosis, obstructive chronic lung disease, and cancer. In other words, having multiple co-morbidities is a reality in older people. Therefore, to develop equations for the elderly is a challenge. In Brazil, the average life expectancy is about 74 years old (4), which is another limiting factor.

Enright et al. (24) derived regression equations from "healthy" adults aged 65 years or older with a smoking history of up to 5 pack-years, but that quit smoking before turning 50. There were 288 individuals, with 82 below the age of 70 years. The results demonstrated that PEF was greater than expected in 10% of the cases. Part of this finding was attributed to the systemic arterial hypertension in the controlled "healthy" subjects. The higher the arterial pressure, the greater the maximum expiratory flow. The influence from arterial pressure was also a finding in the work of Pereira et al. (5), which linked lower FVC and FEV_1_ values to arterial hypertension.

Another analysis included healthy individuals who were physically active. It is well known that physical activity can increase lung volume, especially the practice of water polo, basketball, rowing (e.g., canoeing), handball, and soccer (25,26). The equations developed in this study were generated from a sedentary population. This probably caused the values to be lower in comparison with the other equations.

Brändli et al. (19) and Crapo et al. (15) did not exclude smokers or ex-smokers from their sample. Today, smoking is seen as an unhealthy habit, since it is well-known that it can lead to an acute or chronic reduction in pulmonary function (2,3,27,28).

The place where the exams are carried out also have a relevant effect on pulmonary function. Values generated with field devices generally use pneumotachographs and are corrected or rendered homogeneous by BTPS (body temperature and pressure, saturated) conditions. However, the environmental pollution factor cannot be corrected and it directly interferes with lung function, especially in cities with high pollution levels (29). Equations of Crapo et al. (15) and Knudson et al. (10) featured this factor.

The values used in our equations were standardized by using only one device, by the same technique, taken in the same laboratory during the morning, which differs from all other equations presented and discussed in this article.

In order to properly represent the population being studied, we enrolled the minimum number required. Quanjer et al. (30) considered 100 to be the minimum number per gender required for creating an equation. Similar to the study carried out by Knudson et al. (10) it took many years to obtain the appropriate sample size for our study. Knudson et al. (10) performed a double-triple questionnaire check. We performed a double questionnaire check for morbidity and mortality. The questionnaire by Charlson was also used as a check against the first, which was broader and included questions regarding symptoms. In order to avoid biases regarding "forgotten" diseases or disease denial or impaired respiratory function, chest radiographs were performed in all patients, similarly to Crapo et al. (15), as well as electrocardiograms. These criteria made our sample selection a very rigorous process.

Ethnicity is an important variable of complex identification. In our data, self-declared ethnicity did not differ significantly among the groups from which the reference values were derived. It is understood that ethnicity affects body proportions, such as the Cormic index, which is the relation between the height measured at sitting position (encephalic-trunk height) and standing height. Lung volume would be more correlated with seating height than with standing height (stature). This can occur in up to 53% of African descendants and Caucasian-Americans (31). Our analysis did not confirm differences between ethnicities as it has also been shown in some Brazilian genetic studies (32,33). This might be explained, at least in part, by the broad miscegenation in our population.

Gender can account for up to 30% of pulmonary function variation and the separation of reference equations by gender is common (34). We found differences of up to 31% in respiratory volumes between genders. It is understood that men’s larger lung size also interferes with all the other airway components (35). This partially explains the lower FEV_1_/FVC in men than women, implying that the airways are more subjected to dynamic compression (20).

The choice of using a certain reference equation can result in the characterization of a specific respiratory disorder in some individuals (36). The GLI (13) used a new statistical model (LMS), which is the transformation of metric data into parametric data. The LMS is a method that can equalize errors. It turns nonparametric samples in parametric samples and it has been used in various parts of the world. Thus, the method can merge data from different parts of the world using the interpolation variables. The ERS task force derived equations for reference values using a bank of 160,000 individuals from 72 pulmonary function laboratories in 33 countries. After applying the exclusion criteria, a significant number of 97,759 "healthy," non-smoking individuals, who were 2.5 to 95 years of age with different ethnicities, were included, such as Caucasians, African-Americans, northern and southeastern Asians, Latin Americans, Native Americans, Polynesians, and Arabs. It is very difficult to obtain homogeneous data with such a mixture of numbers and ethnicities. Therefore, such research should be considered a proposal and not an operational standard, since it was not a prospective and controlled study and data were collected in pulmonary function labs with different quality levels. Despite these contradictory facts, the equations were made using the LMS method (13). When we compared the data from the linear equations for men and women, both FVC and FEV_1_ did not show statistical differences. In other words, the LMS method or linear regression should yield similar values, which can be beneficial when using the LMS method for international equations in the future.

The linear regression equations featured similar values for the coefficients, residuals, and asymmetry when compared to the logarithmic equations, including those for respiratory flows, which is also accepted by the Knudson et al. (10) and Hankinson et al. (16) equations.

Studies have suggested that FVC and FEV_1_ are proportional to body size (2,20,37–39). This means that a taller individual, with bigger lungs, would have a greater decrease in pulmonary volumes with age, while smaller individuals would have a smaller decrease (20,38). In our study, however, both men and women showed similar decreases with age.

The methodology used in our study of Brazilian equations was crucial towards the differences in absolute values obtained in the study by Pereira et al. (5), whose research generated higher values than ours. The reference values of Knudson et al. (10) are still valid for the Brazilian population.

The equations derived from the study by Pereira et al. (5) are being used in Brazil. However, the authors had not published spirometry equations for Afro-Brazilian ethnicity. Our findings did not differ between self-defined Afro-Brazilian and non-Afro-Brazilian. Thus, the equations presented can also fill this gap. Another aspect to be noted is that there is a tendency in the literature to qualify the Brazilian ethnicity as the same of other Latin America countries. However, the migratory and colonizing currents among Latin American countries were different, which could interfere in spirometry values.

The coefficient of determinations of the spirometric equations were not close to 1. One of the reasons for this could be that the equations have frequently used the same variables (gender, age, height). One of the main advantages of our study is the use of a simple formula without logarithmic scales. Another important positive aspect is that we provide a new Brazilian equation obtained with a different method than previous studies. As in other countries, such as the United States, lung function laboratories may choose which equation is more suitable.

The LMS model for producing equations can be used in the Brazilian population. One of the characteristics of this method is statistical evolution and the potential to have standard spirometry reference values in the future.

## References

[1] Sunderman FW (1975). Current concepts of "normal values", "reference values", and "discrimination values" in clinical chemistry. Clin Chem.

[2] SBPT (2002). Sociedade Brasileira de Pneumologia e Tisiologia. Diretrizes para Testes de Função Pulmonar. J Pneumol.

[3] Pellegrino R, Viegi G, Brusasco V, Crapo RO, Burgos F, Casaburi R (2005). Standardisation of spirometry. Eur Respir J.

[4] IBGE Brasília: Instituto Brasileiro de Geografia e Estatística [updated 2015 Aug 28; cited 2016 May 3].

[5] Pereira CAC, Sato T, Rodrigues SC (2007). Novos valores de referência para espirometria forçada em brasileiros adultos de raça branca. J Bras Pneumol.

[6] WHO World Health Organization [updated 2016 June; cited 2016 Jul 12].

[7] Charlson M, Szatrowski TP, Peterson J, Gold J (1994). Validation of a combined comorbidity index. J Clin Epidemiol.

[8] Allen SC, Charlton C, Backen W, Warwick-Sanders M, Yeung P (2010). Performing slow vital capacity in older people with and without cognitive impairment - is it useful?. Age Ageing.

[9] Salpeter S, Ormiston T, Salpeter E (2002). Cardioselective beta-blockers for reversible airway disease. Cochrane Database Syst Rev.

[10] Knudson RJ, Lebowitz MD, Holberg CJ, Burrows B (1983). Changes in the normal maximal expiratory flow-volume curve with growth and aging. Am Rev Respir Dis.

[11] Craig CL, Marshall AL, Sjostrom M, Bauman AE, Booth Ml, Ainsworth Be (2003). International Physical Activity Questionnaire: 12-Country Reliability and Validity. Med Sci Sports Exerc.

[12] ATS (1987). American Thoracic Society. Standardization of spirometry - 1987 update. Am Rev Respir Dis.

[13] Quanjer PH, Stanojevic S, Cole TJ, Baur X, Hall GL, Culver BH (2012). ERS global lung function initiative. Multi-ethnic reference values for spirometry for the 3-95-yr age range: the global lung function 2012 equations. Eur Respir J.

[14] Kubota M, Kobayashi H, Quanjer PH, Omori H, Tatsumi K, Kanazawa M (2014). Clinical Pulmonary Functions Committee of the Japanese Respiratory Society. Reference values for spirometry, including vital capacity, in Japanese adults calculated with the LMS method and compared with previous values. Respir Investig.

[15] Crapo RO, Morris AH, Gardner RM (1981). Reference spirometric values using techniques and equipment that meet ATS recommendations. Am Rev Respir Dis.

[16] Hankinson JL, Odencrantz JR, Fedan KB (1999). Spirometric reference values from a sample of the general U.S. population. Am J Respir Crit Care Med.

[17] Pérez-Padilla R, Valdivia G, Muião A, López MV, Márquez MN, Montes de Oca M (2006). Spirometric reference values in 5 large Latin American cities for subjects aged 40 years or over. Arch Bronconeumol.

[18] Falaschetti E, Laiho J, Primatesta P, Purdon S (2004). Prediction equations for normal and low lung function from the Health Survey for England. Eur Respir J.

[19] Brändli O, Schindler C, Künzli N, Keller R, Perruchoud AP (1996). Lung function in healthy never smoking adults: reference values and lower limits of normal of a Swiss population. Chest.

[20] Clausen JL (1982). Pulmonary function testing guidelines and controversies: equipment, methods, and normal values.

[21] Becklake MR (1986). Concepts of normality applied to the measurement of lung function. Am J Med.

[22] MacNee W, Rabinovich RA, Choudhury G (2014). Ageing and the border between health and disease. Eur Respir J.

[23] Barnett K, Mercer SW, Norbury M, Watt G, Wyke S, Guthrie B (2012). Epidemiology of multimorbidity and implications for health care, research, and medical education: a cross-sectional study. Lance.

[24] Enright PL, Adams AB, Boyle PJ, Sherrill DL (1995). Spirometry and maximal respiratory pressure references from healthy Minnesota 65- to 85-year-old women and men. Chest.

[25] Mazic S, Lazovic B, Djelic M, Suzic-Lazic J, Djordjevic-Saranovic S, Durmic T (2015). Respiratory parameters in elite athletes-does sport have an influence?. Rev Port Pneumol.

[26] Durmic T, Lazovic B, Djelic M, Lazic JS, Zikic D, Zugic V, Dekleva M (2015). Sport-specific influences on respiratory patterns in elite athletes. J Bras Pneumol.

[27] SBPT (1996). I Consenso Brasileiro sobre Espirometria. J Pneumol.

[28] Cohen CA, Hudson AR, Clausen JL, Knelson JH (1972). Respiratory symptoms, spirometry, and oxidant air pollution in nonsmoking adults. Am Rev Respir Dis.

[29] da Silva LF, Saldiva SR, Saldiva PH, Dolhnikoff M, Bandeira Científica Project (2012). Impaired lung function in individuals chronically exposed to biomass combustion. Environ Res.

[30] Quanjer PH, Stocks J, Cole TJ, Hall GL, Stanojevic S (2011). Global Lungs Initiative Influence of secular trends and sample size on reference equations for lung function tests. Eur Respir J.

[31] Harik-Khan RI, Muller DC, Wise RA (2004). Racial difference in lung function in African-American and White children: effect of anthropometric, socioeconomic, nutritional, and environmental factors. Am J Epidemiol.

[32] Lins TC, Vieira RG, Abreu BS, Grattapaglia D, Pereira RW (2010). Genetic composition of Brazilian population samples based on a set of twenty eight ancestry informative SNPs. Am J Hum Bio.

[33] Friedrich DC, Genro JP, Sortica VA, Suarez-Kurtz G, de Moraes ME, Pena SD (2014). Distribution of CYP2D6 alleles and phenotypes in the Brazilian population. PLoS One.

[34] Collins DV, Cutillo AG, Armstrong JD, Crapo RO, Kanner RE, Tocino I (1986). Large airway size, lung size, and maximal expiratory flow in healthy nonsmokers. Am Rev Respir Dis.

[35] Martin TR, Castile RG, Fredberg JJ, Wohl ME, Mead J (1987). Airway size is related to sex but not lung size in normal adults. J Appl Physiol.

[36] Vaz Fragoso CA, McAvay G, Van Ness PH, Casaburi R, Jensen RL, MacIntyre N (2015). Phenotype of normal spirometry in an aging population. Am J Respir Crit Care Med.

[37] Lowery EM, Brubaker AL, Kuhlmann E, Kovacs EJ (2013). The aging lung. Clin Interv Aging.

[38] Knudson RJ, Slatin RC, Lebowitz MD, Burrows B (1976). The maximal expiratory flow-volume curve: normal standards, variability, effects of age. Am Rev Respir Dis.

[39] Quanjer PH, Capderou A, Mazicioglu MM, Aggarwal AN, Banik SD, Popovic S (2014). All-age relationship between arm span and height in different ethnic groups. Eur Respir J.

